# Publisher’s Note: Continued Publication of *Infectious Disease Reports* by MDPI

**DOI:** 10.3390/idr12030010

**Published:** 2020-10-16

**Authors:** Franck Vazquez, Shu-Kun Lin, Unai Vicario

**Affiliations:** 1 MDPI, St. Alban-Anlage 66, CH-4052 Basel, Switzerland; lin@mdpi.com; 2 MDPI, Avenida Madrid, 95, 08028 Barcelona, Spain; vicario@mdpi.com

*Infectious Disease Reports* was launched in 2009 and it has been published over the past eleven years by PAGEPress Publications [1]. Professor David M. Aronoff served as the first Editor-in-Chief from 2009 to 2012 [2,3] and the second Editor-in-Chief Dr. Nicola Petrosillo is currently still active in this role [4].

We are excited to take over the publication of *Infectious Disease Reports* from PAGEPress and perpetuate the tradition of this journal. *Infectious Disease Reports* expands well the MDPI portfolio of epidemiology journals, and complements well—just to cite a few—with *International Journal of Environmental Research and Public Health* [5], *Epidemiologia* [6], *Tropical Medicine and Infectious Disease* [7] or *Diseases* [8].

MDPI will publish the fourth quarterly issue of 2020, and thereafter publish four quarterly issues as of 2021. 

Enjoy publishing your work in *Infectious Disease Reports* [9]!
